# Correction: Mineralocorticoid receptor (MR) antagonist eplerenone and MR modulator balcinrenone prevent renal extracellular matrix remodeling and inflammation via the MR/proteoglycan/TLR4 pathway

**DOI:** 10.1042/CS-2024-0302_COR

**Published:** 2024-10-07

**Keywords:** chronic kidney disease, inflammation, mineralocorticoid receptor, renal fibrosis

The authors of the original article “Mineralocorticoid receptor (MR) antagonist eplerenone and MR modulator balcinrenone prevent renal extracellular matrix remodeling and inflammation via the MR/proteoglycan/TLR4 pathway” DOI: 10.1042/CS20240302 would like to correct Table 1 in their paper. The authors state that there is an error in the Na+ (mmol/24h) CKD-HFD Balcin (7) value, which should read “0.10±0.01”, instead of “0.01±0.01”. The requested correction has been assessed and agreed by the Editorial Board.

**Table 1:** Effects of MR blockade on renal collagen deposition

	Sham-HFD (5)	CKD HFD (7)	CKD-HFD Balcin (7)	CKD-HFD Eple (6)
**Creatinine (μM)**	10.5±0.4	25.0±1.7*	27.7±1.5*	27.9±3.7*
**Urea (mM)**	6.96±0.40	18.51±2.97*	18.94±0.72*	16.65±1.65*
**Urine volume (ml)**	0.9±0.1	3.2±0.5*	3.7±0.2*	4.1±0.4*
**Albuminuria (μg/24h)**	13.8±1.2	35.3±7.8*	27.9±3.5	40.5±13.8
**Aldosteronuria(pg/24h)**	1809±560	6920±1735*	7439±1088*	11385±3033*
**Na^+^ (mM)**	155.2±1.5	159.9±1.9	150.4±1.2#	153.3±2.2#
**K^+^ (mM)**	3.8±0.1	3.9±0.2	4.6±0.3*	4.7±0.2*#
**Na^+^ (mmol/24h)**	0.13±0.02	0.17±0.01	0.10±0.01	0.13±0.03
**K^+^ (mmol/24h)**	0.28±0.04	0.38±0.04	0.36±0.03	0.36±0.06
**Ht (%)**	34.8±0.4	25.4±1.4*	29.6±0.5*#	27.8±1.4*#
**Hb (g/dL)**	11.9±0.1	8.6±0.5*	10.1±0.2*#	9.4±0.5*
	* p<0.05 vs Sham-HFD			
	**# p<0.05 vs CKD-HFD**			
**Ht: hematocrit; Hb: Hemoglobin.**				

Physiological parameters of the groups Sham-HFD, CKD-HFD, CKD-HFD Balcin and CKD-HFD Eple measured at the sacrifice. The 24h urine collection for albuminuria, aldosteronuria, natriuria and kaliuria measurements was retrieved one week before sacrifice. Data represent the mean ± SEM. Statistical analysis by one-way ANOVA; with Tukey post-test *P<0.05 *; **P ≤ 0.01; ****P ≤ 0.0001 n = 5-7.

